# Modulation of Symbiont Lipid A Signaling by Host Alkaline Phosphatases in the Squid-Vibrio Symbiosis

**DOI:** 10.1128/mBio.00093-12

**Published:** 2012-05-01

**Authors:** Bethany A. Rader, Natacha Kremer, Michael A. Apicella, William E. Goldman, Margaret J. McFall-Ngai

**Affiliations:** Department of Medical Microbiology and Immunology, University of Wisconsin—Madison, Madison, Wisconsin, USA^a^;; Department of Microbiology, University of Iowa, Iowa City, Iowa, USA^b^; and; Department of Microbiology and Immunology, University of North Carolina, Chapel Hill, North Carolina, USA^c^

## Abstract

The synergistic activity of *Vibrio fischeri* lipid A and the peptidoglycan monomer (tracheal cytotoxin [TCT]) induces apoptosis in the superficial cells of the juvenile *Euprymna scolopes* light organ during the onset of the squid-vibrio symbiosis. Once the association is established in the epithelium-lined crypts of the light organ, the host degrades the symbiont’s constitutively produced TCT by the amidase activity of a peptidoglycan recognition protein (*E. scolopes* peptidoglycan recognition protein 2 [EsPGRP2]). In the present study, we explored the role of alkaline phosphatases in transforming the lipid A of the symbiont into a form that changes its signaling properties to host tissues. We obtained full-length open reading frames for two *E. scolopes* alkaline phosphatase (EsAP) mRNAs (*esap1* and *esap2*); transcript levels suggested that the dominant light organ isoform is EsAP1. Levels of total EsAP activity increased with symbiosis, but only after the lipid A-dependent morphogenetic induction at 12 h, and were regulated over the day-night cycle. Inhibition of total EsAP activity impaired normal colonization and persistence by the symbiont. EsAP activity localized to the internal regions of the symbiotic juvenile light organ, including the lumina of the crypt spaces where the symbiont resides. These data provide evidence that EsAPs work in concert with EsPGRPs to change the signaling properties of bacterial products and thereby promote persistent colonization by the mutualistic symbiont.

## IMPORTANCE

The potential for microbe-associated molecular patterns (MAMPs) to compromise host-tissue health is reflected in the often-used nomenclature for these molecules: lipopolysaccharide (LPS) is also called “endotoxin” and the peptidoglycan monomer is also called “tracheal cytotoxin” (TCT). With constant presentation of MAMPs by the normal microbiota, mechanisms to tolerate their effects have developed. The results of this contribution provide evidence that host alkaline phosphatases (APs) dephosphorylate and inactivate the symbiont MAMP lipid A. As such, APs work in synergy with a peptidoglycan recognition protein, which inactivates symbiont-exported TCT, to alter the symbiont MAMPs and promote persistence of the partnership. Not only may these activities serve to “tame” the MAMPs, but also the resulting products may themselves be important signals in persistent mutualisms. The finding of lipid A modification by APs in an invertebrate mutualism provides evidence that this specific strategy for dealing with symbiotic partners is conserved across the animal kingdom.

## Introduction

Coevolved animal-microbe interactions require specific communication for partner recognition and recruitment, modulation of the symbiont’s environment, and the entry into lifelong, productive partnerships. Microbe-associated molecular patterns (MAMPs) (1), which mediate many such interactions, are microbe-specific biochemical signatures that are recognized by the host through pattern recognition receptors (PRRs). Recent studies of the activity of these molecules, while principally characterizing them in pathogenic associations, have demonstrated that MAMPs and PRRs mediate many facets of the reciprocal interactions of beneficial host-microbe partnerships (2–4). The occurrence of such interactions in both pathogenic and beneficial associations suggests alternative strategies for modulating MAMP/PRR activity that result in the different outcomes of these relationships.

The association between the Hawaiian bobtail squid *Euprymna scolopes* and the luminous marine bacterium *Vibrio fischeri* is an established, natural model for the persistent colonization of animal epithelia by bacterial symbionts (for a review, see reference 5). More recently, this light organ symbiosis has become an emerging model for the study of host innate immune response to MAMPs (4). Bacterial MAMPs, both nonspecific (derived from environmental nonsymbionts) and specific (*V. fischeri* derived), are important players in the early events of the establishment of the symbiosis (1, 6–8).

**FIG 1:**
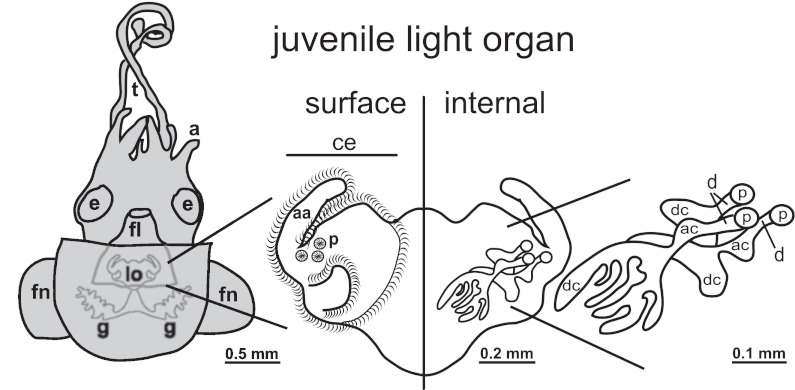
The *E. scolopes* juvenile light organ has a complex three-dimensional morphology. (Left) Diagram of the ventral surface of a juvenile animal. The organs of the animal, including the light organ (lo) and gills (g), are suspended in the center of the animal’s mantle (body) cavity and, in this view, are visible through the translucent ventral mantle. (Center) Enlargement of a bisected organ showing the external surface (left) and the internal crypt environment (right), where the bacterial symbionts reside. (Right) Depiction of the detailed anatomy of the three crypts of one side of the organ. a, arms; aa, anterior appendages; ac, antechambers; ce, ciliated epithelium; d, ducts; dc, deep crypts; e, eyes; fl, funnel; fn, fins; p, pores; t, tentacles.

*V. fischeri* is acquired from the environment in each host generation. Within 6 to 8 h of hatching, symbiont cells colonize the microvillous surfaces of epithelium-lined crypts of the juvenile host (Fig. 1) (for a review, see reference 5), a process that is potentiated by a juvenile-specific field of ciliated cells on the light organ surface (7). The bacteria irreversibly signal light organ morphogenesis at about 12 h following the onset of the association (9), one conspicuous feature of which is the apoptosis-driven regression of the ciliated epithelial surface (10). MAMPs of *V. fischeri*, specifically, lipopolysaccharide (LPS) and peptidoglycan (PGN) derivatives, act in synergy to promote the apoptotic program and light organ morphogenesis (1). The lipid A component of *V. fischeri* LPS signals the characteristic chromatin condensation of early-stage apoptosis (6), and the peptidoglycan monomer (tracheal cytotoxin [TCT]) signals late-stage apoptosis, which involves DNA fragmentation and nuclear loss of a host PRR, *Euprymna scolopes* peptidoglycan recognition protein 1 (EsPGRP1) (8). The completion of this developmental process, while irreversibly triggered at around 12 h, requires 4 to 5 days following initial contact of the host with *V. fischeri* (10). Another conspicuous feature of the symbiosis is a set of host and symbiont diel rhythms, which begin at the first dawn following the onset of the symbiosis, i.e., at about 12 h, and continue through the life of the animal. On a daily basis, the animals vent ~90% of the crypt contents into the environment each day at dawn and the bacterial symbionts regrow over the day to fill the crypt spaces (11–13). The nocturnal animal emerges at dusk from its diurnal resting place in the sand to use the luminescence of the dense population of symbionts as camouflage while it forages at night in the water column.

Once *V. fischeri* populations are established in the crypts, they continuously present MAMPs to the associated host cells. As such, it has been predicted that the host has mechanisms for modulating presentation of these perturbing molecules. In a recent study (14), it was demonstrated that EsPGRP2, another squid host PRR, is secreted into the deep crypts of symbiotic animals and has the specific PGRP-type amidase activity required to degrade the TCT molecule. This study also showed that secretion into the crypts did not begin until after the 12-h TCT-requiring signal for morphogenesis induction. Such a “taming” of PGN by the host in a mutualism is not unprecedented. In *Drosophila melanogaster*, the amidase activity of PGRP-LB in the hemolymph detoxifies the PGN derivatives presented by the intestinal microbiota (15).

Along with TCT, the concentration of *V. fischeri* LPS is predicted to increase in the crypts as symbiont cell density increases, which may also drive host mechanisms for the modulation of LPS activity. A recent elegant study in zebra fish demonstrated that alkaline phosphatases (APs) detoxify LPS presented by gut symbionts (16). The APs do so by the cleavage of the phosphate groups from the sugar backbone of the lipid A moiety, a process that was demonstrated first in host responses to bacterial pathogens (17–19); this dephosphorylation of the lipid A molecule renders it incapable of inducing an inflammatory response in host tissues. In zebra fish and other vertebrates, the intestine-specific AP isozyme is localized to the apical brush border of the epithelial lining at microvillar tips and is enriched in luminal vesicles derived from the microvillar membrane (20, 21). As the squid epithelium in the light organ crypts is ultrastructurally similar to that of the gut and the physical relationship between host and symbiont cells is analogous, a similar strategy may be used to control the activity of symbiont lipid A in the squid-vibrio partnership.

In this study, we sought to determine whether *E. scolopes* alters the properties of *V. fischeri* LPS through the activity of APs. Two AP transcripts were annotated in an expressed sequence tag (EST) library created from juvenile *E. scolopes* light organs (22), suggesting the presence of AP activity in association with symbiont-containing tissues. We present results implicating *E. scolopes* alkaline phosphatases (EsAPs) as active participants in the events of both the early colonization and the maintenance of the symbiosis. These data provide evidence that AP dephosphorylation of LPS is not only a requirement of vertebrate organ systems harboring complex consortia but also a conserved mechanism for the establishment and maintenance of mutualistic symbioses across the animal kingdom.

## RESULTS

### *E*. *scolopes* expresses two alkaline phosphatase transcripts in the light organ.

**FIG 2:**
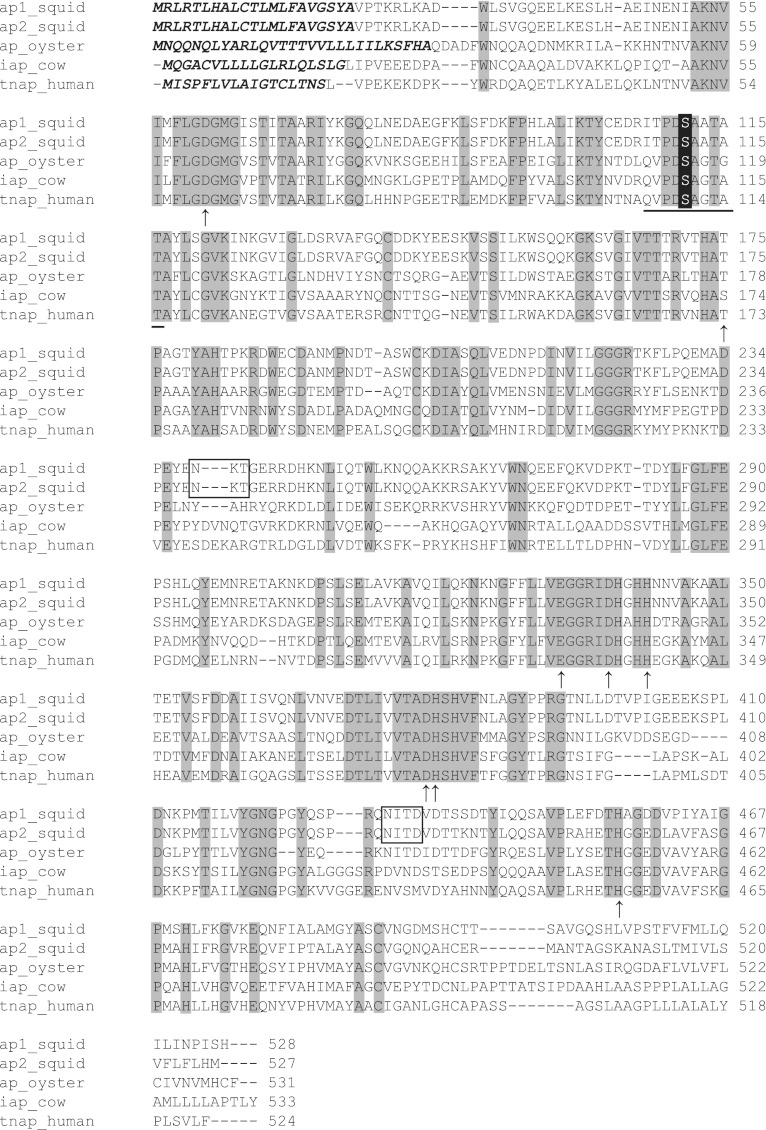
Clustal W sequence alignment of EsAP1 and EsAP2 with proteins in the AP family. Bold and italicized text indicates a signal peptide, identical amino acids are shaded gray, and proposed metal-binding sites are identified with arrows. The active site is underlined, and the conserved serine that is required for activity is outlined in black. Open boxes indicate predicted N-glycosylation sites. The proteins used in the alignment are squid EsAP1 (ap1; accession number AER46069), EsAP2 (ap2; accession no. AER46070), alkaline phosphatase from oyster (ap; accession no. AAV69062.1), intestinal alkaline phosphatase from cow (iap; accession number NP_776412), and tissue (nonspecific) from human (tnap; accession number NP_000469.3).

**FIG 3:**
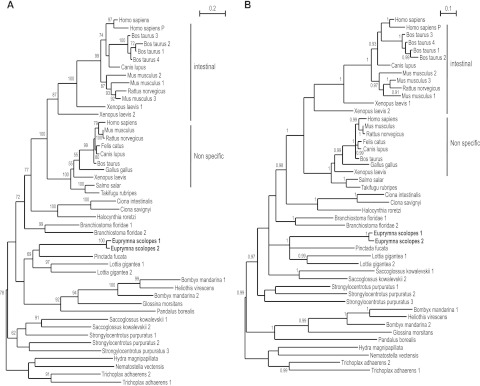
Phylogenetic analysis of the amino acid sequences of EsAP1 and EsAP2. Sequences were chosen to provide broad representation across the animal kingdom (see Table S1 in the supplemental material). (A) Maximum likelihood inference phylogeny based on alkaline phosphatase sequences. Support values of >50 obtained after 100 bootstrap replicates are shown at corresponding nodes. (B) Bayesian inference phylogeny based on alkaline phosphatase sequences. Bayesian posterior probabilities of >0.9 are shown at corresponding nodes. Positions of *E. scolopes* sequences are shown in bold. *Euprymna scolopes* 1, EsAP1; *Euprymna scolopes* 2, EsAP2.

An EST library created from juvenile *E. scolopes* light organ mRNA (22) had two partial alkaline phosphatase (AP) sequences. Rapid amplification of cDNA ends (RACE) of both sequences revealed that *esap1* (open reading frame [ORF], 1,585 bp) and *esap2* (ORF, 1,581 bp) are identical isoforms up to position 1386, where they differ, possibly as a result of alternative splicing. This variation in the 3′ end results in two isoforms that are 90% identical to one another in amino acid sequence, 44 to 53% identical to eukaryotes in the nonredundant database (BLASTp), and 24% identical to *Escherichia coli* (BLASTp). Analysis of the alignment of the EsAPs (Fig. 2) revealed a conserved signal sequence consisting of the first 21 amino acids, as well as conservation of the active-site residues I106 to T116, residues critical for metal binding (D61, T175, E333, D338, H342, D379, H380, H456), and essential N-glycosylation sites (N239 to T241 and N432 to D435) (23, 24). These two sequences have differences in the carboxy-terminal region of the protein, including different amino acids and gaps at alternative locations. In phylogenetic analyses, the EsAPs branch together and cluster with the other mollusk APs. Other closely related invertebrates form a group with the mollusks (Fig. 3) (see also Table S1 in the supplemental material).

### *esap1* and *esap2* are differentially expressed in squid tissues.

**FIG 4:**
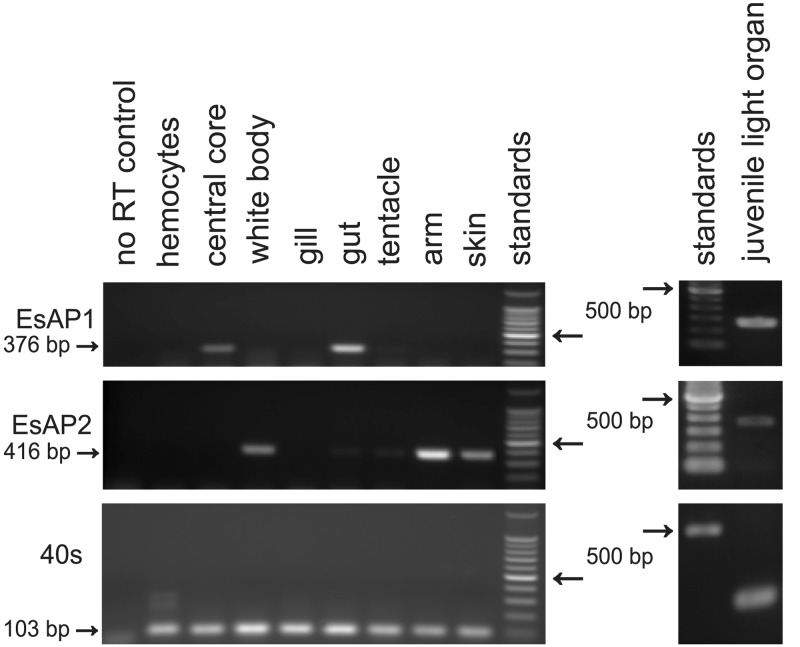
Endpoint PCR analysis of *esap1* and *esap2* in adult squid tissues and juvenile light organ. The RT reactions for each tissue were performed using 1 µg of total RNA. PCRs were run for 35 cycles, and 10 μl of PCR mixture was loaded into each lane. The 40S ribosomal gene was used as a housekeeping gene control. A control reaction where no reverse transcriptase was added was run to assess genomic contamination. Results are representative of three independent extractions.

Because transcripts encoding different alkaline phosphatase isoforms vary in tissue expression in other animals, we sought to determine whether the two *esap* transcripts are differentially expressed in host squid tissues. We performed endpoint semiquantitative reverse transcriptase PCR (RT-PCR) on cDNA amplified by poly(dT) primer from RNA extracted from tissues (Fig. 4). Our results revealed the presence of *esap1* mRNA in guts and bacterium-containing central epithelial light organ cores (central cores) of adult animals, which can be dissected cleanly from adult light organs, and whole light organs of juvenile animals, which will contain both juvenile central core and gut. *esap2* was highly expressed in other tissues, specifically, the skin, arm, and white body (hematopoietic organ). It had lower but detectable levels of expression in the tentacle and gut. Expression in the central core, gill, and mature circulating hemocytes, which are blood cells produced by the white body, was undetectable.

### EsAPs have a pH optima of ~8.

**FIG 5:**
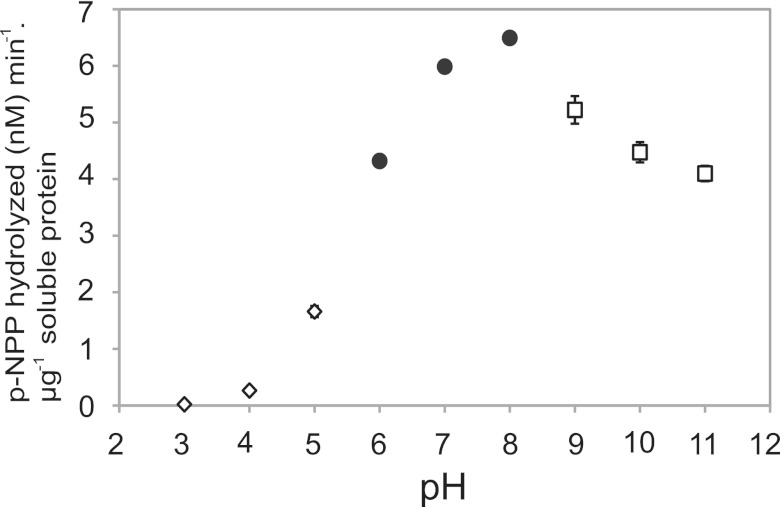
pH optima of the light organ EsAPs. The values represent phosphate activity in the central core extracts relative to buffer pH. ◊, reactions in sodium acetate buffer (pK_a_, 4.76); ●, reactions in Tris-HCl buffer (pK_a_, 8.06); □, reactions in sodium carbonate buffer (pK_a_, 10.35). Bars indicate that standard errors are present, although some are too small to be distinguished from the data point; *n* = 5 technical replicates.

We sought to determine the pH optimum of the putative AP activity in the light organ for two reasons: (i) to confirm that the observed activity is due to alkaline and not acid phosphatases and (ii) because diel fluctuations in crypt pH have been implicated in the control of the symbiosis (11). We pooled the extracted total soluble proteins from the light organ central cores of four adult animals and measured activity across a range from pH 3 to 11. A peak of activity occurred at pH 8.0, with activity decreasing with increasing acidity or alkalinity (Fig. 5). No activity peak at the lower pHs was detected, suggesting that the central core of the light organ has low or no acid phosphatase activity.

### Regulation of AP activity begins between 12 and 24 h following onset of the colonization process and exhibits a daily rhythm.

**FIG 6:**
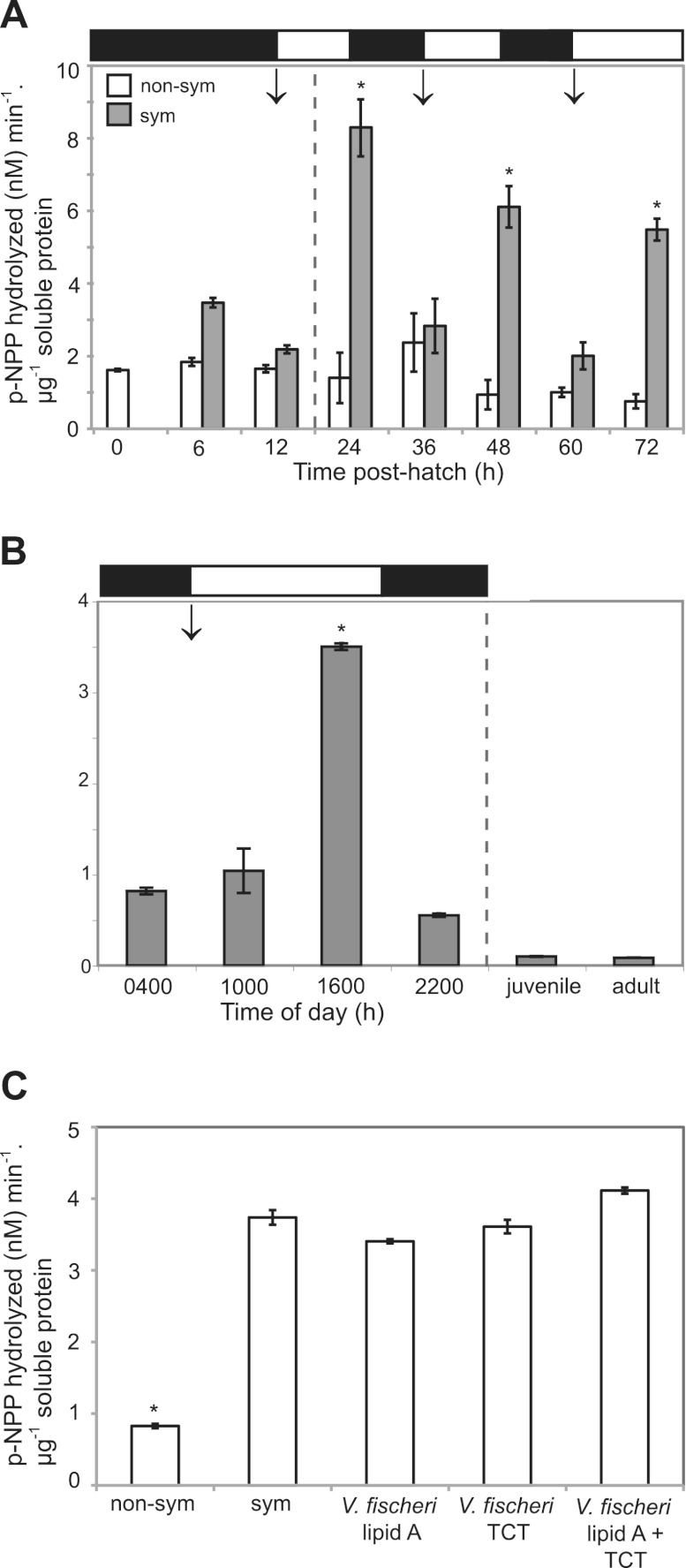
Characterization of EsAP activity in juvenile and adult light organs. (A) EsAP activity measured as *p*-NPP hydrolysis in age-matched nonsymbiotic (non-sym) and symbiotic (sym) juvenile light organ extracts over the first 3 days following hatching. Each bar represents the average (±standard error) EsAP activity in three biological replicates (*n* = 15 light organs/replicate; for each biological replicate, 5 technical replicates were performed). *, treatments that are significantly different from nonsymbiotic values (two-way analysis of variance with *post hoc* pairwise comparisons, *P* < 0.001). Dashed line, change in time scale of the *x* axis. (B) Left of dashed line, EsAP activity in adult light organ central cores. Each bar represents EsAP activity in three biological replicates; each biological replicate is two central cores from a single animal. Each bar represents the average (±standard error) EsAP activity in three biological replicates (*n* = 15 light organs/replicate; for each biological replicate, 5 technical replicates were performed). *, treatment that is significantly different from all other treatments (one-way analysis of variance with *post hoc* pairwise comparisons, *P* < 0.001). Bars above the graphs in panels A and B indicate day (white) and night (black). Arrows indicate dawn. Right of dashed line, EsAP activity in combined juvenile light organ samples and combined adult central core samples incubated with 10 ng ml^−1^ levamisole. Each bar represents the average (±standard error) of 5 technical replicates. (C) EsAP activity in juvenile animals after incubation with either *V. fischeri* or *V. fischeri*-derived MAMPs. Each bar represents the average (±standard error) EsAP activity in two biological replicates (*n* = 12 light organs/replicate; for each biological replicate, 5 technical replicates were performed). *, treatment that is significantly different from all other treatments (one-way analysis of variance with *post hoc* pairwise comparisons, *P* < 0.001).

To define the patterns of AP activity during the onset of the symbiosis and to determine whether the activity exhibited a diel rhythm, we isolated total soluble protein from light organs at various time points over the first 72 h of the association, as well as over the day-night cycle in adult animals, and assayed for total AP activity (Fig. 6). In juveniles (Fig. 6A), over the first 12 h of the association, activity levels were relatively low and were not significantly different between nonsymbiotic and symbiotic light organs. At 24, 48, and 72 h, i.e., around dusk of the daily rhythm, EsAP activity was significantly higher in the organs of symbiotic animals than in those of nonsymbiotic animals; at 12, 36, and 60 h, i.e., around dawn, no significant difference in AP activity was detected between nonsymbiotic and symbiotic animals. To determine whether the diel patterns observed in the juveniles persist in the adult animal and to resolve the timing more precisely over the day-night cycle, we assayed for EsAP activity on total soluble protein extracted from light organ central cores dissected from adult animals at 4 time points over the day-night cycle (Fig. 6B). EsAP activity was relatively low at all time points except 1600 h, when activity was 3 to 4 times higher; this time corresponds to a time when the light organ is full of *V. fischeri* and light production of the symbionts is under induction. This activity assay cannot distinguish between the AP isoforms, but the semiquantitative RT-PCR suggested that activity is primarily due to EsAP1. No EsAP activity was detected from the host membrane fraction or from soluble and membrane protein fractions of extracts of cultured bacteria (data not shown). This *in vitro* EsAP activity in both juvenile and adult animals was inhibited to background levels by 10 ng ml^−1^ levamisole, a specific, reversible, noncompetitive inhibitor of nonintestinal alkaline phosphatases (Fig. 6B) (25).

Because LPS and other bacterial MAMPs, including TCT, are active in many aspects of the symbiosis, we sought to determine if symbiosis-induced AP activity was due to exposure to symbiont-derived MAMPs. In these experiments, we incubated juvenile animals with *V. fischeri* MAMPs. The presumed EsAP substrate, *V. fischeri* lipid A, as well as TCT alone or the combination of lipid A and TCT, induced EsAP activity to a level similar to that induced by exposure to the symbiont (Fig. 6C).

### The AP inhibitor levamisole compromises normal colonization of the juvenile light organ.

**FIG 7:**
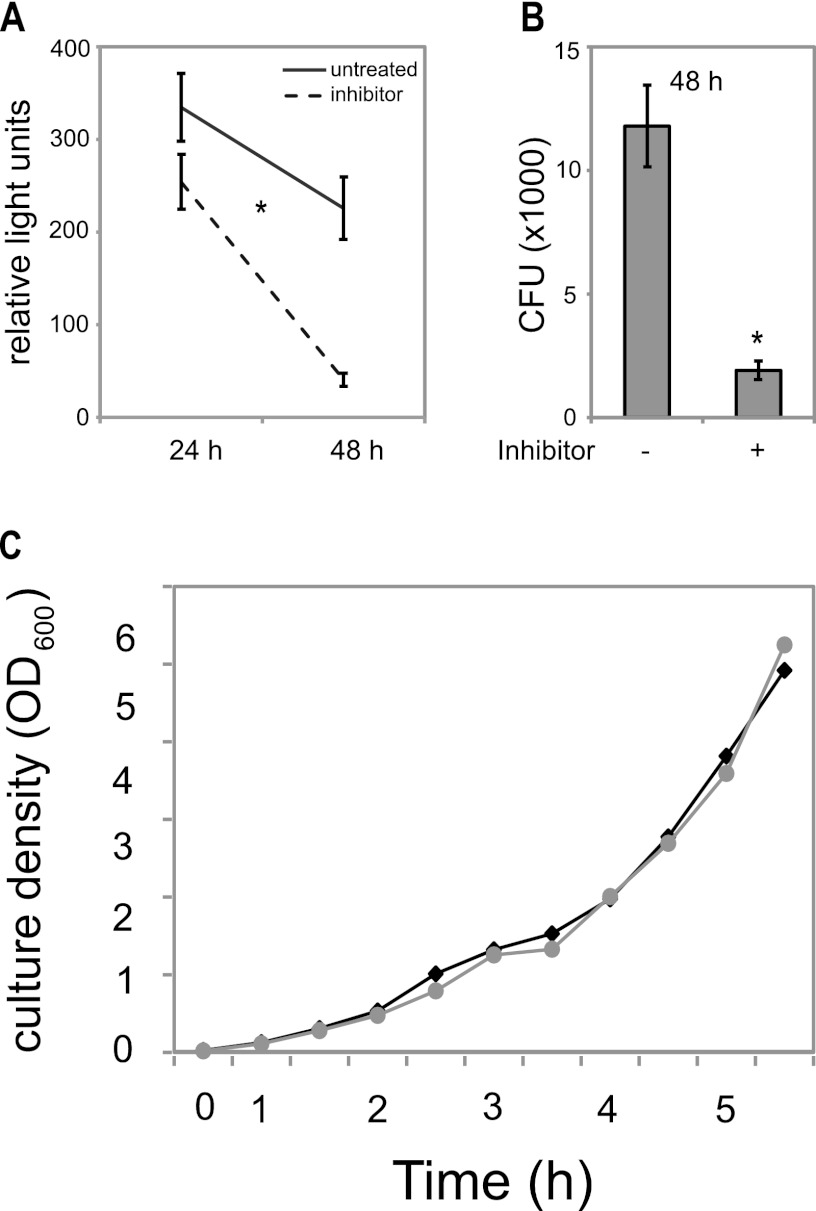
Inhibition of endogenous alkaline phosphatase *in vivo*. (A) Effect of 10 ng ml^−1^ levamisole on the luminescence of juvenile animals at 24 and 48 h of colonization. Bars, average relative light units per treatment (±standard error; *n* = 18 animals per treatment). *, inhibitor-treated animal significantly different from untreated animal (linear regression, *t* test on slopes, *P* < 0.05). (B) Effect of inhibitor on symbiont number (CFU) in the light organ of animals at 48 h of colonization. Average relative light units per treatment (±standard error; *n* = 18 animals per treatment). *, inhibitor-treated animal significantly different from untreated animal (*t* test on slopes with unequal variances, *P* < 0.05). (C) Effect of inhibitor on ES114 growth in culture measured at OD_600_. Black line, growth in LBS medium alone; gray line, growth in LBS medium supplemented with 10 ng ml^−1^ levamisole.

The patterns of regulation described above suggested that EsAPs are important to the symbiosis. To test this hypothesis, we assayed for the effects of the AP inhibitor levamisole on early stages of the association. We incubated hatchling animals for 48 h in the presence of *V. fischeri* ES114 alone or ES114 with 10 ng ml^−1^ levamisole and measured luminescence at 24 and 48 h and CFU/light organ at 48 h (Fig. 7A and 7B). At 24 h, the luminescence output of inhibitor-treated animals was ~75% of that of untreated animals (Fig. 7A). At 48 h, both luminescence and CFU/light organ were lower by more than 80% in treated animals (Fig. 7A and 7B). Per-cell luminescence of the bacteria was not affected by the inhibitor, nor was growth rate under culture conditions affected (Fig. 7C). These data suggest that active alkaline phosphatase is required to achieve and maintain a normal symbiosis.

### EsAP activity localizes to the internal regions of the light organ.

**FIG 8:**
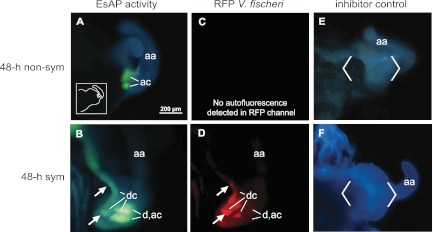
Localization of EsAP activity in intact juvenile light organs. Images of fluorescence in 48-h nonsymbiotic and 48-h symbiotic light organs. (A and B) Detection of EsAP activity using ELF-97 substrate. The inset in panel A outlines the area of the light organ depicted in the images. (C and D) Localization of *V. fischeri* producing red fluorescent protein (RFP). Arrows indicate where the AP activity and bacteria colocalize. (E and F) Light organs incubated in ELF-97 substrate with the AP inhibitor levamisole (10 ng ml^−1^) as a control. Angle brackets indicate the region where AP activity is present in the absence of inhibitor. Bar, 200 µm; aa, anterior appendage; ac, antechamber; d, ducts; dc, deep crypts.

Because the results showed that EsAP activity is high in total light organ extracts when symbiont density is high, we sought to determine the specific location of EsAP activity. Fixed light organs from 48-h symbiotic and nonsymbiotic animals were incubated with a substrate, ELF-97, which has been used in the study of alkaline phosphatase activity (26) and which produces a fluorescent precipitate when dephosphorylated. Nonsymbiotic and symbiotic juvenile light organs had intense signal in the duct and antechamber, although these regions appeared brighter in symbiotic animals than in nonsymbiotic animals (Fig. 8A and 8B). However, fluorescence in the crypts was detected only in symbiotic animals (Fig. 8A to 8D); as the bacteria had no detectable AP activity (see Materials and Methods), the substrate fluorescence signal was host associated. Light organs were also incubated (i) in the presence of substrate containing the AP inhibitor levamisole to confirm that the observed signal was specific to AP (Fig. 8E and 8F) or (ii) in detection buffer alone to ensure that the solution itself did not induce autofluorescence of light organ tissues (data not shown). Levamisole treatment abrogated the fluorescence signal of ELF-97, confirming that the signal was associated with AP activity.

### Alkaline phosphatase treatment of *V*. *fischeri* lipid A compromises the ability of lipid A to induce normal apoptosis in developing light organ tissues.

**FIG 9:**
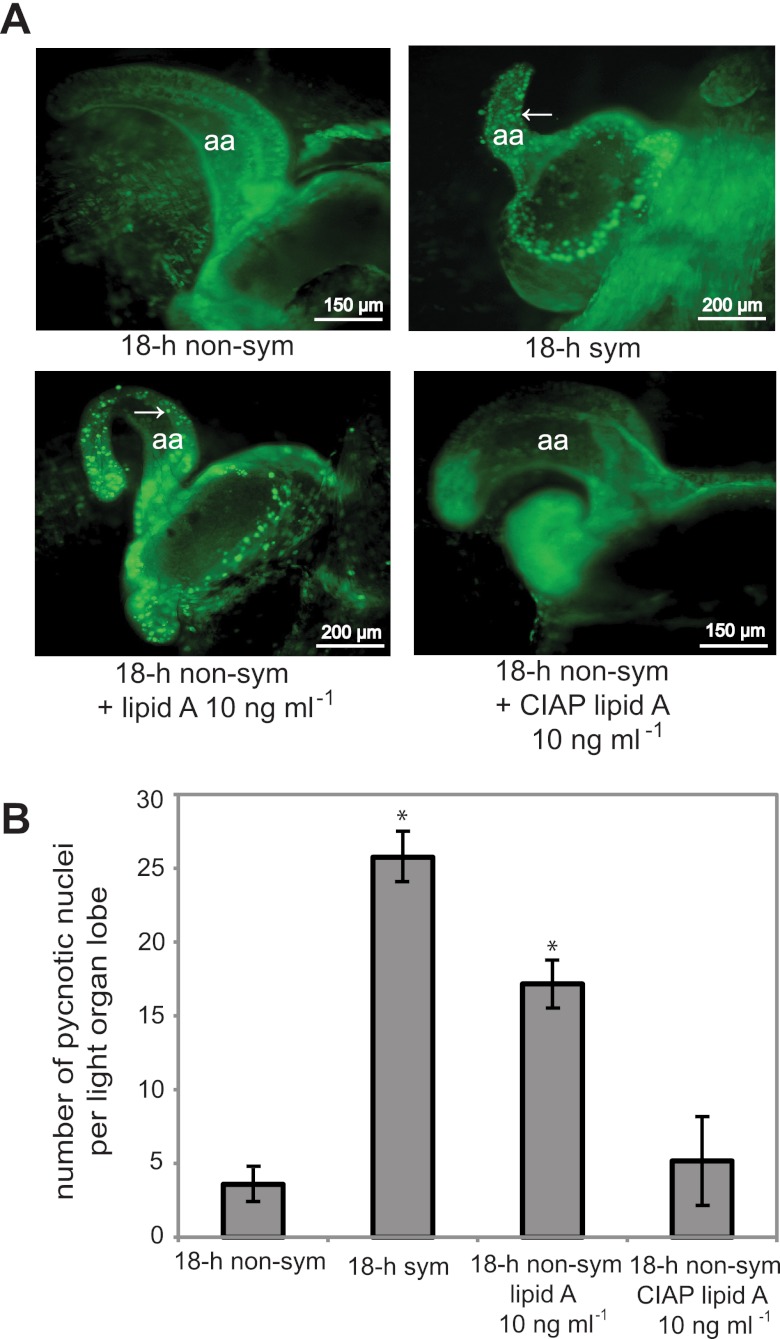
Effect of CIAP treatment of lipid A on its ability to induce juvenile light organ apoptosis. (A) Fluorescence micrographs of light organs that had been incubated in filter-sterilized artificial seawater alone (non-sym) or with ES114 (sym), non-sym animal light organs incubated in FSASW plus lipid A, or non-sym animal light organs incubated in FSASW plus CIAP-treated lipid A for 18 h and stained with acridine orange (green) to visualize pycnotic nuclei, which appear as regions of punctate staining across the diffusely stained field of epithelial cells (e.g., arrow). Bars, 150 or 200 µm. (B) The number of pycnotic nuclei per anterior appendage of the juvenile light organ (*n* = 12 per treatment). Bars, average (±standard deviation); *, treatments that are significantly different from other treatments but not each other (one-way analysis of variance with *post hoc* pairwise comparisons, *P* < 0.001). aa, anterior appendage, the region used for enumeration of pycnotic nuclei.

To determine whether alkaline phosphatase dephosphorylation can render *V. fischeri* lipid A nonreactive, we took advantage of the previous finding that *V. fischeri* lipid A is required for entry into early-stage apoptosis (chromatin condensation) of the superficial epithelial cells of the juvenile light organ (6). We incubated hatchling animals for 18 h in filter-sterilized artificial seawater with *V. fischeri* lipid A, lipid A that had been treated with calf intestinal alkaline phosphatase (CIAP), *V. fischeri* ES114 as a positive control for chromatin condensation, or seawater alone as a negative control. Whereas lipid A caused early-stage apoptosis at levels indistinguishable from those of symbiont-colonized juveniles, animals exposed to CIAP-treated lipid A had levels indistinguishable from those of animals exposed to seawater alone (Fig. 9).

## DISCUSSION

The assignment of the proteins characterized in this study as alkaline phosphatases is supported by sequence characteristics and phylogenetic relationships to other alkaline phosphatases. In addition, support for AP activity in light organ tissue extracts includes the pH optima, inhibition by levamisole, and fluorescence induction of ELF-97 and the ability to compromise the activity of lipid A. Although the molecular data suggest at least two EsAP isoforms in the host squid, EsAP1 and EsAP2, the dominant isoform in the light organ is EsAP1.

The data presented provide evidence that the host animal controls the presentation of AP in symbiotic tissues both during early development and over the day-night cycle. The molecular exchange between host and symbiont is carefully orchestrated so that the apoptotic loss of the superficial ciliated field, which is principally induced by presentation of lipid A and TCT, is triggered only following a successful colonization of the light organ crypts. Specifically, we found that AP activity is maintained at relatively low levels until after the 12-h irreversible lipid A-mediated signal for the onset of early apoptosis is delivered by the symbionts. This finding mirrors our results with the EsPGRP2-mediated degradation of the other morphogen, TCT, which is required for the cells to enter late-stage apoptosis (14). After this initial period of low AP activity and EsPGRP2 protein levels, the cellular and molecular evidence suggests that both proteins are secreted into the crypt spaces throughout the life of the host, taming the MAMPs constitutively presented by *V. fischeri*. Additional support for the importance of AP in crypt homeostasis is provided by results of experiments using the inhibitor levamisole: the inhibition of AP activity, as early as 24 h following onset of the symbiosis, compromises persistence, suggesting that AP activity is essential for the maintenance of the association.

Analysis of AP activity over the diel cycle suggested that the enzyme activity is high in tissue extracts in the late afternoon and early evening hours, when the *V. fischeri* population density and the luminescence of the cells are at their peak. This time is also coincident with the period during which the animal host is actively foraging and using the luminescence of the bacterial symbionts as camouflage. These data suggest that host tissues are rendered insensitive to signaling of symbiont lipid A by AP dephosphorylation of the molecule during this critical period. In the late evening, the levels of AP activity decrease; this time corresponds to a period during which symbiont chitin fermentation is predicted to acidify the crypt spaces, which would result in inactivation of the AP. In the hours just before the dawn venting, symbiont population densities are still high, but AP levels are then low. This time corresponds to the hours before dawn, when the signature of host gene expression provides evidence for widespread perturbation of host cytoskeleton (11). As such, low AP activity may allow the lipid A to once again become a strong signal to host cells. Thus, in the squid host tissues during the hours around dawn, LPS may be signaling change in the form and function of the host cytoskeleton as it does in many mammalian systems (see, e.g., reference 27). Around dawn, the crypt cells are effaced, i.e., both the microvilli and the apical surfaces of the cells are shed into the crypt lumen. In the hours following dawn, these crypt cells repolarize, regaining their complex brush borders. Whether the lipid A signals the effacement, the regrowth, or both processes remains to be determined.

The patterns of the relationship between AP activity and host biology reflect symbiont transcriptomic data over the day-night cycle (11). These data suggest that the symbionts have a metabolic diel rhythm: they anaerobically respire the shed host membranes, which is a pH-neutral process, over the day and anaerobically ferment chitin, which is an acidifying metabolism, in the hours before dawn. Thus, when the crypt environment is likely to have a neutral pH (over the day into early evening), both the activity of resident AP, which is optimal at neutral pH, and its concentration are at peak; conversely, when the crypt environment is predicted to be acidic (in the hours before dawn), both the activity of the AP and its concentration are low.

The inability of EsAP-treated *V. fischeri* lipid A to cause the typical symbiont-induced apoptosis suggests that the symbiont lipid A is dephosphorylated by the AP. Derived LPS structures for most bacterial species indicate that the lipid A moiety is typically diphosphorylated with phosphate groups decorating the glucosamine disaccharide. The structure of wild-type *V. fischeri* lipid A has recently been derived and shown to be highly unusual (28). Relevant here is the finding that, in addition to the typical phosphorylation on the sugar residues, the fatty acid acyl chains carry a phosphoglycerol moiety, which is unprecedented among the LPS structures thus far derived. Whether the EsAPs are active in the dephosphorylation of all phosphorylated sites and, if so, what that would mean to the overall function of the LPS remain to be examined.

Our findings suggest that the role of alkaline phosphatases in the regulation of animal-microbe associations is a shared characteristic of both vertebrates and invertebrates. Intestinal AP (IAP) has been recently and intensively studied in vertebrates, both in health and in disease; it participates in the maintenance of gut immune homeostasis (29), and recent evidence has demonstrated that IAP influences the development of intestinal diseases, such as inflammatory bowel disease and Crohn’s disease (30). The occurrence of vertebrate IAP at the microvillar brush border and in membrane-bound vesicles that are transported to the lumen (20, 21, 31) suggests that vertebrate IAP is likely presented to the gut microbiota in much the same way that light organ crypt AP is presented to *V. fischeri*. Cell culture studies support the idea that IAP compromises the activity of LPS that would be presented by gut symbionts; specifically, IAP overexpression in HT-29 cells blocked LPS-mediated RelA/p65 translocation to the nucleus (32). As with all animals, *E. scolopes* expresses the genes encoding proteins of the NF-κB pathway (33).

The most compelling studies showing that vertebrate gut-consortial interactions require AP activity for homeostasis come from experimental research with germfree zebra fish. These investigations have demonstrated that, as in the squid-vibrio system light organ crypts, APs act on LPS presented by mutualistic symbionts. Introduction of LPS into the zebra fish gut initiates cytokine and IAP production, increasing IAP activity and initiating a negative-feedback loop in which IAP-dephosphorylated LPS decreases cytokine production (16). In addition, inhibition of IAP in zebra fish either by l-phenylalanine or by an IAP splice-blocking morpholino-oligonucleotide resulted in hypersensitivity to LPS.

Taken together, the data presented here for the role of APs in the establishment and homeostasis of the squid-vibrio symbiosis provide a prime example of how selection pressure over evolutionary time can result in intricate tuning of the interaction between coevolved partners in a mutualistic symbiosis. In a broader view, we provide evidence that eukaryotic hosts are able to detect and respond to bacterial MAMPs as beneficial signals, and perhaps also as toxins, by temporal and spatial regulation of proteins, such as the alkaline phosphatases and the PGRPs, that will modulate the MAMP activity. As such, these studies provide insight into the mechanisms underlying how mutualistic symbionts can make peace, not war, using the same molecular language as that of pathogens.

## MATERIALS AND METHODS

### General procedures.

Adult *E. scolopes* animals were collected, transported, and maintained as previously described (34). All animals were anesthetized in 2% ethanol in seawater or filter-sterilized artificial seawater (FSASW) prior to sacrifice. Juvenile squid were colonized, and colonization was confirmed as previously described (8).

Juvenile squid that were exposed to various reagents were placed at one animal per well in 12-well non-tissue-culture microtiter dishes. Under these conditions, animals were exposed to 10 µM *V. fischeri* TCT, 10 ng ml^−1^ *V. fischeri* lipid A, 10 ng ml^−1^ *V. fischeri* dephosphorylated lipid A, or 10 µM levamisole as an alkaline phosphatase inhibitor (Acros Organics, Geel, Belgium). Lipid A and TCT were prepared as previously described (1, 6) to ensure that there was no cross-contamination between these MAMPs.

All reagents were purchased from Sigma-Aldrich (St. Louis, MO) unless otherwise specified. Confocal microscopy was performed on a Zeiss Axioplan 2 microscope (Carl Zeiss MicroImaging Inc., Thornwood, NY). Pictures were taken using a Canon Powershot S5 IS camera (Canon Global, Tokyo, Japan).

### RACE and sequence analysis.

Two 3′ EsAP sequences were identified in a previously constructed EST database derived from cDNA libraries of juvenile light organs (22). To obtain both full-length EsAP sequences, 5′ and 3′ RACE was performed on total RNA extracted from whole juvenile animals. RACE fragments were cloned using the TOPO TA cloning kit (Invitrogen, Carlsbad, CA) for sequencing. All kits were used according to the manufacturers’ instructions. Sequencing was performed at the DNA Sequencing Laboratory, University of Wisconsin—Madison. Sequence alignment was carried out using ClustalW (http://www.ebi.ac.uk/Tools/msa/clustalw2/). The signal peptides were identified using SignalP (http://www.cbs.dtu.dk/services/SignalP/), and the O- and N-glycosylation sites were identified using Expasy NetOGlyc 3.1 and NetNGlyc 1.0 servers (http://www.cbs.dtu.dk/services) (35, 36). Additional essential residues were defined by comparing the sequences to those of other well-characterized APs (23, 24).

Alignment of alkaline phosphatase sequences used in phylogenetic analyses was generated using MUSCLE (37) and manually checked using SeaView (38). Ambiguously aligned regions were removed, and the following regions were selected for tree reconstruction: 31 to 45, 49 to 142, 145 to 211, 213 to 256, 261 to 300, 303 to 305, 308 to 405, 411 to 423, and 429 to 492. Maximum likelihood reconstruction was performed using PhyML 3.0 (39) with the WAG+Γ+I model, and 100 bootstrap replicates were conducted for support estimation. Bayesian reconstruction was performed using PhyloBayes 2.3 (40) under the site-heterogeneous CAT model. Two chains were run for at least 64,000 cycles, and the first 1,000 cycles were removed as burn-in.

### RT-PCR.

Tissues were dissected from the animals and stored at −80°C in RNAlater (Ambion, Austin, TX). Total RNA from all samples was extracted using TRIzol reagent (Invitrogen, Carlsbad, CA) from an average of 30 juvenile light organs (0.05 to 0.10 µg total RNA/juvenile light organ) and from the mantle epithelium, arm, tentacle, gut, gill, white body (hematopoietic organ), central core, and mature circulating hemocytes of adult animals. Contaminating DNA was removed using the Turbo DNA-Free kit (Ambion, Austin, TX). All RT reactions were performed using 1 µg of total RNA, SMART Moloney murine leukemia virus (MMLV) reverse transcriptase (RT) enzyme (Clontech, Mountain View, CA), oligo(dT), and reaction mixtures were incubated for 1.5 h at 42°C. All reactions were performed with a no-RT control. Specific primers used to amplify the two alkaline phosphatases for RT-PCR were EsAPF (5′ TGGTGAACGTCGAGGACACATTGA 3′), used for amplification of both EsAP1 and EsAP2; EsAP1R (5′ CAGGATGCGTAACCCATTGCAAGA 3′), for amplification of EsAP1; and EsAP2R (5′ ATTAGCCATCCTCTCGCAATGTGC 3′), for amplification of EsAP2. As a loading control, primers 40SF (5′ AATCTCGGCGTCCTTGAGAA 3′) and 40SR (5′ GCATCAATTGCACGACGAGT 3′) were used to amplify the 40S ribosomal subunit protein S19. PCRs used the following program: 3 min at 94°C and 35 cycles of 30 s at 94°C, 30 s at 55°C, and 45 s at 72°C, with a final extension of 10 min at 72°C. Products were separated on a 1% agarose gel, stained with ethidium bromide, and visualized using a FluorChem 8400 imager (Alpha Innotech Corp., San Leandro, CA).

### Quantification, inhibition, and localization of AP in host tissues.

To quantify AP activity in light organ tissues from adult and juvenile squid and determine its source (i.e., host or bacterial) and regulation, tissues were extracted and homogenized in phosphate-buffered saline (PBS) (50 mM sodium phosphate, 0.1 M sodium chloride, pH 7.4) plus a protease inhibitor cocktail (1%, vol/vol) (Sigma-Aldrich; catalog no. P8340). The homogenate was centrifuged at 12,000 × *g* for 20 min at 4°C, and the supernatant fluid was recovered for analysis. To determine whether the symbiont-containing pellet had detectable AP activity, the pellet was washed 4 times with PBS plus protease inhibitors and then subjected to 4 freeze-thaw cycles over a dry-ice ethanol bath followed by 2 min of sonication. This extract was also spun at 12,000 × *g* for 20 min at 4°C, and the supernatant fluid was recovered for analysis. The protein concentration of the fractions was quantified by the Bradford assay. In determinations of AP activity, 1 µg of soluble protein was added to 50 µl of 100 mM Tris-HCl, pH 8.0, with 50 µM *p*-nitrophenol phosphate (*p*-NPP; New England Biolabs, Ipswich, MA) for a final concentration of 20 µg ml^−1^ protein. This mixture was incubated for 30 min at room temperature, and then absorption was measured at 405 nm. The data were calculated as nanomoles of *p*-NPP hydrolyzed to *p*-NP (*p*-nitrophenol) ⋅ min^−1^ ⋅ µg^−1^ protein. Cultured *V. fischeri* cells equivalent to the numbers in host tissues were also similarly treated, and AP activity was also measured in these extracts. To determine whether *V. fischeri* MAMPs induced EsAP activity in juvenile light organs, we exposed animals to FSASW, 3,000 CFU ⋅ ml^−1^ *V. fischeri*, 10 µM *V. fischeri* TCT, 10 ng ⋅ ml^−1^ *V. fischeri* lipid A, or a combination of both lipid A and TCT and incubated them for 18 h, at which time AP activity in the light organ was measured.

To determine whether a well-characterized AP inhibitor, levamisole, inhibited EsAP activity, we first determined an optimal concentration of 10 ng ml^−1^ levamisole. Hatchling animals were exposed to FSASW ± 10 ng ml^−1^ levamisole in seawater (nonsymbiotic) or seawater containing 5 × 10^3^ CFU ⋅ ml^−1^ of *V. fischeri* ES114 ± 10 ng ⋅ ml^−1^ levamisole (symbiotic) for 24 or 48 h. AP activity was measured as described above. Luminescence was determined at both 24 and 48 h to determine whether the inhibitor affected the symbiotic state. At 48 h, the animals were sacrificed, homogenized, and dilution plated on LBS (Luria-Bertani salt medium) agar plates to quantify the number of *V. fischeri* cells per animal.

To determine whether levamisole is inhibitory to bacterial growth, we performed a growth curve in which a starting culture of *V. fischeri* ES114 at an optical density at 600 nm (OD_600_) of 0.05 was grown at 28°C in LBS medium alone or LBS medium supplemented with 10 ng ml^−1^ levamisole. OD_600_ readings were taken approximately every 0.5 h for 6 h. In addition, because this inhibitor can also affect neural activity through nicotinic acetylcholine receptors, we assayed the effects of the inhibitor on squid, including swimming behavior and ventilation rates, which are controlled by the nervous system. No differences with the addition of the inhibitor were detected in these activities (data not shown).

To determine whether pH affects AP activity, total soluble protein extracted from central cores from 4 adult animals was prepared as described above. AP activity was measured in the presence of the following buffers: 100 mM sodium acetate buffer at pH 3, 4, or 5; Tris-HCl buffer at pH 6, 7, or 8; and sodium carbonate buffer at pH 10 and 11.

To localize AP activity, hatchling, 24-h, and 48-h symbiotic and nonsymbiotic animals were first fixed in 4% paraformaldehyde in marine PBS (mPBS), and light organs were dissected and permeabilized as previously described (8), followed by a 10-min rinse in PBS. Visualization and localization of alkaline phosphatase activity were performed using the ELF-97 endogenous phosphatase detection kit (Invitrogen, Eugene, OR). Briefly, ELF-97 phosphatase substrate was diluted 1:20 in detection buffer to a final volume of 200 µl, to which were added 6 juvenile light organs/treatment/trial (*n* = 3 trials/treatment). Following a 1-h incubation, the light organs were visualized by microscopy under UV excitation. Control light organs were incubated either in detection buffer alone or in the presence of substrate containing 5 mM levamisole to inhibit the AP activity.

### Detection of chromatin condensation.

To determine the influence of AP phosphorylation state on the ability of LPS to induce early-stage apoptosis (6), juvenile animals were incubated with 10 ng ml^−1^ *V. fischeri* lipid A, 10 ng ml^−1^ *V. fischeri* lipid A dephosphorylated with 0.1 unit calf intestinal alkaline phosphatase (CIAP; Promega, Madison, WI), *V. fischeri* cells (as described above under “General procedures”), or FSASW alone (*n* = 12 for each treatment). At 18 h, the animals were anesthetized and incubated for 1 min in 5 ng ml^−1^ acridine orange in FSASW. The mantles and funnels were then removed to expose the light organ for visualization. The number of pycnotic nuclei was quantified in one anterior appendage of each juvenile squid.

To prepare dephosphorylated lipid A for these experiments, 100 µg of *V. fischeri* lipid A was incubated with 1 unit CIAP for 1 h at 37°C in a protocol modified from reference 16. The lipid A was then heated to 70°C to heat inactivate the CIAP. Nontreated lipid A was exposed to the same temperature regime to control for possible temperature effects on the lipid A molecule, and lipid A was exposed to heat-inactivated CIAP to confirm that the presence of protein alone or the buffer containing the CIAP did not affect lipid A activity.

### Nucleotide sequence accession numbers.

Nucleotide sequence accession numbers were as follows: EsAP1, AER46069; EsAP2, AER46070.

## SUPPLEMENTAL MATERIAL

Table S1 Accession numbers for genes used in phylogenetic analysis. Table S1, XLSX file, 0.1 MB.

## References

[1] Koropatnick TA 2004 Microbial factor-mediated development in a host-bacterial mutualism. Science 306:1186–1188 15539604 10.1126/science.1102218

[2] Round JL Mazmanian SK 2009 The gut microbiota shapes intestinal immune responses during health and disease. Nat. Rev. Immunol. 9:313–323 19343057 10.1038/nri2515 PMC4095778

[3] Eberl G Boneca IG 2010 Bacteria and MAMP-induced morphogenesis of the immune system. Curr. Opin. Immunol. 22:448–454 20580214 10.1016/j.coi.2010.06.002

[4] McFall-Ngai M Nyholm SV Castillo MG 2010 The role of the immune system in the initiation and persistence of the *Euprymna scolopes–Vibrio fischeri* symbiosis. Semin. Immunol. 22:48–53 20036144 10.1016/j.smim.2009.11.003 PMC3992935

[5] McFall-Ngai M 2008 Host-microbe symbiosis: the squid-vibrio association—a naturally occurring, experimental model of animal/bacterial partnerships. Adv. Exp. Med. Biol. 635:102–112 18841707 10.1007/978-0-387-09550-9_9

[6] Foster JS Apicella MA McFall-Ngai MJ 2000 *Vibrio fischeri* lipopolysaccharide induces developmental apoptosis, but not complete morphogenesis, of the *Euprymna scolopes* symbiotic light organ. Dev. Biol. 226:242–254 11023684 10.1006/dbio.2000.9868

[7] Nyholm SV Stabb EV Ruby EG McFall-Ngai MJ 2000 Establishment of an animal-bacterial association: recruiting symbiotic *vibrios* from the environment. Proc. Natl. Acad. Sci. U. S. A. 97:10231–10235 10963683 10.1073/pnas.97.18.10231 PMC27829

[8] Troll JV 2009 Peptidoglycan induces loss of a nuclear peptidoglycan recognition protein during host tissue development in a beneficial animal-bacterial symbiosis. Cell. Microbiol. 11:1114–1127 19416268 10.1111/j.1462-5822.2009.01315.x PMC2758052

[9] Doino JA McFall-Ngai MJ 1995 A transient exposure to symbiosis-competent bacteria induces light organ morphogenesis in the host squid. Biol. Bull. 189:347–355 10.2307/1542152 29244576

[10] Montgomery MK McFall-Ngai M 1994 Bacterial symbionts induce host organ morphogenesis during early postembryonic development of the squid *Euprymna scolopes*. Development (Cambridge) 120:1719–1729 10.1242/dev.120.7.1719 7924980

[11] Wier AM 2010 Transcriptional patterns in both host and bacterium underlie a daily rhythm of anatomical and metabolic change in a beneficial symbiosis. Proc. Natl. Acad. Sci. U. S. A. 107:2259–2264 20133870 10.1073/pnas.0909712107 PMC2836665

[12] Graf J Ruby EG 1998 Host-derived amino acids support the proliferation of symbiotic bacteria. Proc. Natl. Acad. Sci. U. S. A. 95:1818–1822 9465100 10.1073/pnas.95.4.1818 PMC19196

[13] Nyholm SV McFall-Ngai MJ 1998 Sampling the light-organ microenvironment of *Euprymna scolopes*: description of a population of host cells in association with the bacterial symbiont *Vibrio fischeri*. Biol. Bull. 195:89–97 9818359 10.2307/1542815

[14] Troll JV 2010 Taming the symbiont for coexistence: a host PGRP neutralizes a bacterial symbiont toxin. Environ. Microbiol. 12:2190–2203 21966913 10.1111/j.1462-2920.2009.02121.x PMC2889240

[15] Zaidman-Rémy A 2006 The *Drosophila* amidase PGRP-LB modulates the immune response to bacterial infection. Immunity 24:463–473 16618604 10.1016/j.immuni.2006.02.012

[16] Bates JM Akerlund J Mittge E Guillemin K 2007 Intestinal alkaline phosphatase detoxifies lipopolysaccharide and prevents inflammation in zebrafish in response to the gut microbiota. Cell Host Microbe 2:371–382 18078689 10.1016/j.chom.2007.10.010 PMC2730374

[17] Verweij WR 2004 Protection against an *Escherichia coli*-induced sepsis by alkaline phosphatase in mice. Shock 22:174–179 15257092 10.1097/01.shk.0000132485.05049.8a

[18] Tuin A 2006 On the role and fate of LPS-dephosphorylating activity in the rat liver. Am. J. Physiol. Gastrointest. Liver Physiol. 290:G377–G385 16223948 10.1152/ajpgi.00147.2005

[19] Bol-Schoenmakers M 2010 Intestinal alkaline phosphatase contributes to the reduction of severe intestinal epithelial damage. Eur. J. Pharmacol. 633:71–77 20132812 10.1016/j.ejphar.2010.01.023

[20] Alpers DH Zhang Y Ahnen DJ 1995 Synthesis and parallel secretion of rat intestinal alkaline phosphatase and a surfactant-like particle protein. Am. J. Physiol. 268:E1205–E1214 7611397 10.1152/ajpendo.1995.268.6.E1205

[21] McConnell RE 2009 The enterocyte microvillus is a vesicle-generating organelle. J. Cell Biol. 185:1285–1298 19564407 10.1083/jcb.200902147 PMC2712962

[22] Chun CK 2006 An annotated cDNA library of juvenile *Euprymna scolopes* with and without colonization by the symbiont *Vibrio fischeri*. BMC Genomics 7:154–164 16780587 10.1186/1471-2164-7-154 PMC1574308

[23] Kim EE Wyckoff HW 1990 Structure of alkaline phosphatases. Clin. Chim. Acta 186:175–187 2178807 10.1016/0009-8981(90)90035-q

[24] Nilsen IW Øverbø K Olsen RL 2001 Thermolabile alkaline phosphatase from northern shrimp (*Pandalus borealis*): protein and cDNA sequence analyses. Comp. Biochem. Physiol. B Biochem. Mol. Biol. 129:853–861 11435140 10.1016/s1096-4959(01)00391-8

[25] Van Belle H 1976 Alkaline phosphatase. I. Kinetics and inhibition by levamisole of purified isoenzymes from humans. Clin. Chem. 22:972–976 6169

[26] Navarro-Ródenas A Morte A Pérez-Gilabert M 2009 Partial purification, characterisation and histochemical localisation of alkaline phosphatase from ascocarps of the edible desert truffle *Terfezia claveryi* Chatin. Plant Biol. (Stuttg.) 11:678–685 19689775 10.1111/j.1438-8677.2008.00172.x

[27] Moriez R 2005 Myosin light chain kinase is involved in lipopolysaccharide-induced disruption of colonic epithelial barrier and bacterial translocation in rats. Am. J. Pathol. 167:1071–1079 16192642 10.1016/S0002-9440(10)61196-0 PMC1603678

[28] Phillips NJ 2011 The lipid A from *Vibrio fischeri* lipopolysaccharide: a unique structure bearing a phosphoglycerol moiety. J. Biol. Chem. 286:21203–21219 21498521 10.1074/jbc.M111.239475 PMC3122182

[29] Malo MS 2010 Intestinal alkaline phosphatase preserves the normal homeostasis of gut microbiota. Gut 59:1476–1484 20947883 10.1136/gut.2010.211706

[30] Tuin A 2009 Role of alkaline phosphatase in colitis in man and rats. Gut 58:379–387 18852260 10.1136/gut.2007.128868

[31] Eliakim R Mahmood A Alpers DH 1991 Rat intestinal alkaline phosphatase secretion into lumen and serum is coordinately regulated. Biochim. Biophys. Acta 1091:1–8 1671644 10.1016/0167-4889(91)90213-h

[32] Goldberg RF 2008 Intestinal alkaline phosphatase is a gut mucosal defense factor maintained by enteral nutrition. Proc. Natl. Acad. Sci. U. S. A. 105:3551–3556 18292227 10.1073/pnas.0712140105 PMC2265168

[33] Goodson MS 2005 Identifying components of the NF-kappaB pathway in the beneficial *Euprymna scolopes–Vibrio fischeri* light organ symbiosis. Appl. Environ. Microbiol. 71:6934–6946 16269728 10.1128/AEM.71.11.6934-6946.2005 PMC1287678

[34] Montgomery MK McFall-Ngai M 1993 Embryonic development of the light organ of the sepiolid squid *Euprymna scolopes* berry. Biol. Bull. (Woods Hole) 184:296–308 10.2307/1542448 29300543

[35] Emanuelsson O Brunak S von Heijne G Nielsen H 2007 Locating proteins in the cell using TargetP, SignalP and related tools. Nat. Protoc. 2:953–971 17446895 10.1038/nprot.2007.131

[36] Julenius K Mølgaard A Gupta R Brunak S 2005 Prediction, conservation analysis, and structural characterization of mammalian mucin-type O-glycosylation sites. Glycobiology 15:153–164 15385431 10.1093/glycob/cwh151

[37] Edgar RC 2004 MUSCLE: multiple sequence alignment with high accuracy and high throughput. Nucleic Acids Res. 32:1792–1797 15034147 10.1093/nar/gkh340 PMC390337

[38] Gouy M Guindon S Gascuel O 2010 SeaView version 4: a multiplatform graphical user interface for sequence alignment and phylogenetic tree building. Mol. Biol. Evol. 27:221–224 19854763 10.1093/molbev/msp259

[39] Guindon S Gascuel O 2003 A simple, fast, and accurate algorithm to estimate large phylogenies by maximum likelihood. Syst. Biol. 52:696–704 14530136 10.1080/10635150390235520

[40] Lartillot N Lepage T Blanquart S 2009 PhyloBayes 3: a bayesian software package for phylogenetic reconstruction and molecular dating. Bioinformatics 25:2286–2288 19535536 10.1093/bioinformatics/btp368

